# Sawtooth fetal heart rate pattern associated with a favorable neurological outcome in an infant: a case report

**DOI:** 10.1186/s13256-019-2170-0

**Published:** 2019-07-25

**Authors:** Satoshi Ohira, Sakura Yamanaka, Ryoichi Asaka, Hirofumi Ando, Chiho Fuseya, Norihiko Kikuchi, Tsutomu Miyamoto, Makoto Kanai, Tanri Shiozawa

**Affiliations:** Department of Obstetrics and Gynecology, Shinshu University School of Medicine, 3-1-1 Asahi, Matsumoto, 390-8621 Japan

**Keywords:** Sawtooth fetal heart rate pattern, Fetal heart rate monitoring, Neurological sequelae, Case report

## Abstract

**Background:**

The sawtooth fetal heart rate pattern is rare, and has been reported as a possible indicator of neurological sequelae in newborns. However, we observed this fetal heart rate pattern in an infant with normal neurological function.

**Case presentation:**

A 29-year-old primigravida Japanese woman presented to our hospital at 40 weeks and 1 day of gestation with marked vaginal bleeding. Since admission, fetal heart rate tracing consistently demonstrated a sawtooth-like pattern. There were 3–4 oscillations per minute, and their amplitude was 30–40 beats per minute. An emergency cesarean section was performed because of non-reassuring fetal status. Evidence of placental abruption was not observed. The newborn was a male weighing 2936 g, with an Apgar score of 1 and 3 at 1 minute and 5 minutes, respectively. The infant received brain cooling, but was discharged uneventfully. A follow-up examination at age 3 years demonstrated no developmental restriction.

**Conclusion:**

Although the Apgar score of the newborn was low, the infant had no neurological sequelae. Thus, the sawtooth fetal heart rate pattern may not be linked to *in utero* irreversible fetal central nervous system injury.

## Background

Antenatal and intrapartum application of electronic fetal heart rate (FHR) monitoring for the evaluation of fetal condition are of common use in developed countries. The efficacy of electronic FHR monitoring during labor is judged by its ability to decrease complications, such as neonatal seizures, cerebral palsy, or intrapartum fetal death, while minimizing the need for unnecessary obstetric interventions [1, 2]. Despite well-designed basic science investigations into the physiology underlying standard FHR patterns (variability, accelerations, and decelerations), these observations have not translated into measurable positive impacts on newborn outcomes but have contributed significantly to the cesarean delivery rate [3–7]. Recently, Andrikopoulou and Vintzileos [8] reported three cases of documented central nervous system (CNS) injury with a characteristic FHR sawtooth-like pattern. However, we observed this FHR pattern in an infant with normal neurological function. Here we report a case of sawtooth FHR pattern associated with a favorable neurological outcome in an infant.

## Case presentation

**Fig. 1:**
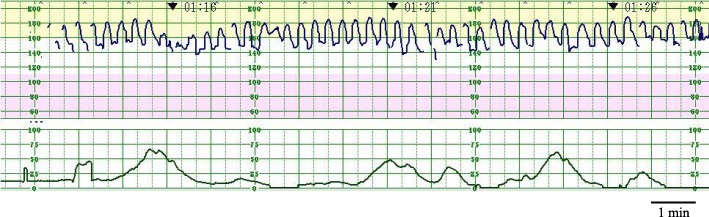
Sawtooth pattern tracings with indeterminate baseline

**Fig. 2:**
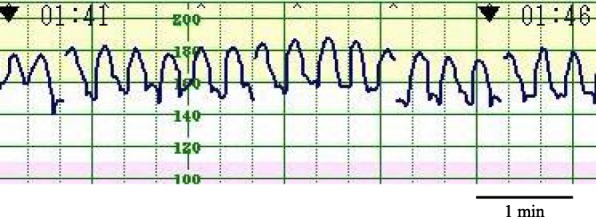
There are 3–4 sharp oscillations per minute, and the amplitude is 30–40 beats per minute

A 29-year-old primigravida Japanese woman presented to our hospital at 40 weeks and 1 day of gestation with marked vaginal bleeding. The posterior placenta had been low-lying, but had migrated to the upper uterine segment in the third trimester. Since admission, FHR tracing consistently demonstrated a sawtooth-like pattern with indeterminate baseline (Fig. 1). There were 3–4 sharp oscillations per minute, and their amplitude was 30–40 beats per minute (bpm) (Fig. 2). Although fetal movement was slightly observed, only a small amount of amniotic fluid was noted by ultrasonography. The middle cerebral artery peak systolic velocity of the fetus was 100 cm/second (1.55 multiples of the median); therefore, we initially suspected fetal anemia.

Emergency cesarean section was performed because of non-reassuring fetal status. Evidence of placental abruption was not observed. The newborn was a male weighing 2936 g, with an Apgar score of 1 and 3 at 1 minute and 5 minutes, respectively. The umbilical artery cord pH was not available because the artery collapsed. The newborn was not severely anemic, with a hemoglobin level of 13.3 g/dl. The venous blood pH was 6.860 and base excess was − 21.9. Sarnat staging for hypoxic-ischemic encephalopathy [9] of this newborn was grade II moderate. Therefore, the infant received brain cooling for 72 hours from 4 hours after birth. No abnormal findings were detected by brain magnetic resonance imaging performed at 13 days after birth, and the infant was discharged uneventfully. A follow-up examination including DENVER II Developmental Screening Test [10] at age 1, 2, and 3 years demonstrated no developmental restriction.

## Discussion

The sawtooth FHR pattern is rare, and is sometimes confused with sinusoidal pattern [8]. This FHR pattern is characterized by the following: (1) three to five sawtooth-like sharp oscillations per minute; (2) amplitude > 20 bpm; and (3) unstable or indeterminate baseline [8]. On the other hand, true sinusoidal FHR pattern is characterized by the following: (1) stable baseline FHR of 120–160 bpm; (2) amplitude of 5–15 bpm; (3) frequency of 2–5 cycles per minute; (4) fixed or flat short-term variability; (5) oscillations of the sinusoidal wave form above and below a baseline; and (6) no area of normal FHR variability or reactivity [11, 12]. The FHR pattern in our case demonstrated 3–4 oscillations per minute, amplitude of 30–40 bpm, and indeterminate baseline; therefore, the FHR pattern in our case is consistent with the above-described features of the sawtooth FHR pattern.

Other than true sinusoidal FHR pattern, another conventional undulating FHR pattern may be due to pseudo-sinusoidal FHR pattern [11, 13]. Pseudo-sinusoidal FHR patterns include all patterns in which undulatory waveforms, or regular FHR baseline oscillations of constant amplitude, alternate with episodes of normal baseline variability or activity [13]. Murphy *et al.* observed 230 pseudo-sinusoidal FHR patterns in labor, and classified 219 into minor (amplitude 5–15 bpm) and 11 into intermediate (amplitude 16–24 bpm) [13]. Major pseudo-sinusoidal patterns (amplitude > 24 bpm) were not observed [13]. Before the sawtooth FHR pattern was proposed, our case might be considered to be a major pseudo-sinusoidal pattern because of large amplitude.

Andrikopoulou and Vintzileos reported three cases of sawtooth FHR pattern due to *in utero* fetal CNS injury [8]. In their first case, there was a sudden onset of sawtooth FHR pattern during labor. The Apgar score after a vaginal delivery was 9 and 9 at 1 minute and 5 minutes, respectively. Magnetic resonance imaging of the newborn, which was performed because of facial twitches, showed infarction of the left cerebral hemisphere because of a middle cerebral artery thrombosis. The approximate timing of the fetal stroke coincided with the abrupt onset of the sawtooth FHR pattern in labor. In their second case, the patient was admitted with sawtooth FHR pattern. Emergency cesarean section was performed and the Apgar score was 0 and 3 at 1 minute and 5 minutes, respectively. An electroencephalogram was abnormal, and the infant received brain cooling but unfortunately died. The third case had sawtooth FHR pattern during the second stage of labor, and the Apgar score was 3 and 6 at 1 minute and 5 minutes, respectively. A cranial ultrasound of the neonate revealed grade II intraventricular hemorrhage.

In our case, the Apgar score of the newborn was low, but the infant had no neurological sequelae. Although the reason why the sawtooth FHR pattern appeared is unclear, we speculate that transient fetal asphyxia was caused by circulatory failure of the placenta due to marked hemorrhage from placental margin.

## Conclusions

This is the first report of sawtooth FHR pattern without fetal CNS injury. Thus, this FHR pattern may not be linked to *in utero* irreversible fetal CNS injury. Further studies are needed to clarify the pathology underlying sawtooth FHR pattern.

## Data Availability

Not applicable.
